# Case report: Acquired neurotrophic tyrosine receptor kinase inhibitor resistance in a patient with pancreatic neuroendocrine carcinoma receiving entrectinib

**DOI:** 10.3389/fonc.2022.1031396

**Published:** 2023-01-10

**Authors:** Wen-Chi Wu, Ming-Huang Chen

**Affiliations:** ^1^ Department of Oncology, Taipei Veterans General Hospital, Taipei, Taiwan; ^2^ Center of Immuno-Oncology, Department of Oncology, Taipei Veterans General Hospital, Taipei, Taiwan; ^3^ Division of Hematology, Department of Medicine, Taipei Veterans General Hospital, Taipei, Taiwan; ^4^ School of Medicine, National Yang Ming Chiao Tung University, Taipei, Taiwan

**Keywords:** entrectinib, neurotrophic tropomyosin receptor kinase (NTRK), pancreatic neuroendocrine carcinoma (PanNEC), liquid biopsy, repotrectinib

## Abstract

Pancreatic neuroendocrine carcinoma (panNEC) is a rare disease. The rearrangements of neurotrophic tropomyosin receptor kinase (NTRK) genes are oncogenic. And in the existed literatures, the prevalence of NTRK3 was only 0.1% in neuroendocrine tumors. NTRK inhibitor was approved for refractory and recurrence NTRK fusion-positive solid tumors did not respond to standard treatment. We described a patient with panNEC who was confirmed to have ETV6-NTRK3 fusion gene by liquid biopsy. The patient initially responded well to entrectinib, a first-generation NTRK inhibitor, but developed resistance with two acquired NTRK3-G623R and NTRK3-G623E mutations detected by a second liquid biopsy. Kirsten rat sarcoma vial oncogene (KRAS) K117N mutation was found initially but became undetectable after resistance. This was the first report demonstrating the novel agent, entrectinib, used for the NTRK3-fusion gene found by the liquid biopsy in panNEC. Our report provides evidence of not only the effectiveness but also the acquired resistance of entrectinib. Also, we highlighted the potential role of genomic sequencing after entrectinib failure. Furthermore, liquid biopsy should be considered if acquiring tissue from the patient is challenging. Further studies regarding NTRK inhibitors in panNEC were needed.

## 1 Introduction

Neuroendocrine carcinoma (NEC), regardless of the organ of origin, has serious outcomes and limited effective treatments.^1^ Personalized oncogenetic or carcinogenetic molecular targets are crucial for improved disease control.^2^ In 2017, Scarpa et al. proposed several possible pathways for neuroendocrine tumors (panNETs) pathogenesis, including the impairment of deoxyribonucleic acid repair, alternation of chromosome remodeling, activation of mammalian target of rapamycin (mTOR) signaling with subsequent autophagy, and triggering of telomere exhaustion.^3^

Neurotrophic tropomyosin receptor kinase (NTRK) genes encode the tropomyosin receptor kinase (TRK) proteins. The fusion of TRK genes is oncogenic and leads to transphosphorylation in the TRK domain and alternation of the RAS-MAPK, mTOR, and PLCγ-PLC pathways.^1,4^ Sigal et al. retrospectively studied 2,417 patients with all subtypes of neuroendocrine tumors, and reported that the prevalence of NTRK3 rearrangements was only 0.1% (six patients).^5^

Entrectinib, a first-generation NTRK inhibitor, was approved by the United States Food and Drug Administration (FDA) in August 2019 for the treatment of TRK fusion-positive solid tumors without known resistances and without suitable alternative treatments.^6^ The approval was based on three clinical trials: ALKA-372-001, STARTRK-1, and STARTRK-2, which enrolled resistant solid tumor patients harboring NTRK fusions.^6^ The overall response rate of the drug was 57%, and 68% of responders had a duration of response of over 6 months.^6^

Here, we describe the case of a patient with panNEC refractory to multiple lines of standard treatment, whose liquid biopsy of blood exhibited ETV6-NTRK3 fusion and who responded to entrectinib with later resistance attribute to two acquired NTRK3-G623R and NTRK3-G623E mutations. An informed consent form for publication of this report was provided and signed.

## 2 Case presentation

**Figure 1:**
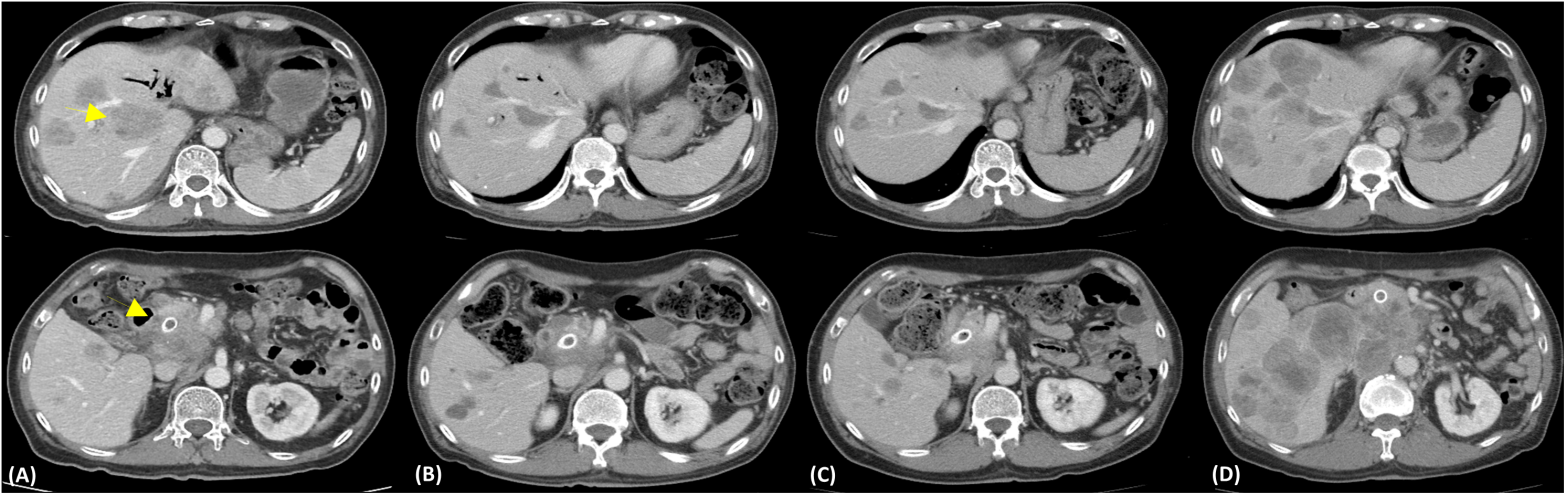
Representative axial computed tomography images. **(A)** Image depicting the disease status before liquid biopsy, with an ill-defined heterogeneous mass about 5.4 cm at the pancreatic head (dotted arrows) and multiple liver metastases 9arrow) **(B)** Subsequent imaging following 3 months of entrectinib treatment (600 mg/day) illustration the resolution of the pancreatic tumor (sized 4.2 cm) and liver metastases **(C)** After 6 months of entrectinib with a further resolution of the tumors. The pancreatic tumor sized only 3.8 cm **(D)** Disease progression (pancreatic tumor sized 7.4 cm) and increased number of liver metastases.

A 62-year-old man presented with jaundice, and in Apirl 2019, was diagnosed as having pancreatic head NEC with encasement of the main portal vein, multiple liver metastases, and lymphadenopathies, stage IV. The disease exacerbated after multiple lines of immune-chemotherapy: the atezolizumab, etoposide, and cisplatin combination; the fluorouracil, leucovorin, irinotecan, and oxaliplatin regimen; gemcitabine with nab-paclitaxel; dacarbazine with fluorouracil; and ipilimumab with nivolumab. Follow-up computed tomography (CT) in August 2020 indicated disease progression with the pancreatic tumor sized 5.4 cm and the largest hepatic metastatic tumor sized 4.2 cm, and lymphadenopathies (**Figure 1A**).

**Figure 2:**
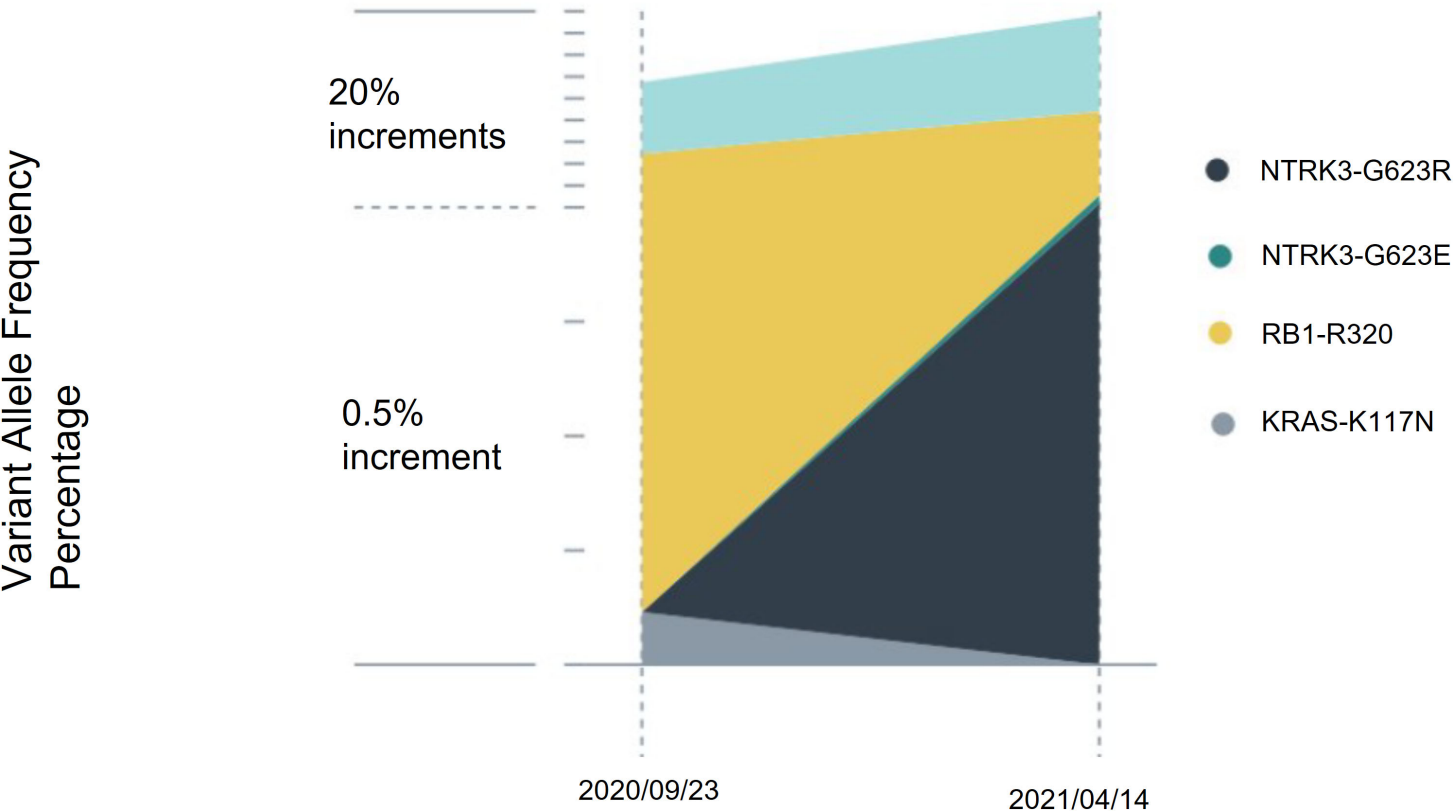
Serial cell-free DNA analysis. Circulating tumor DNA was assessed before entrectinib treatment and at treatment failure. After 8 months of entrectinib, the variation allele frequency of ETV6-NTRK3 decreased, and the RAS mutation became undetectable. However, NTRK3-G623R and NTRK3-G623E mutations emerged.

**Figure 3:**
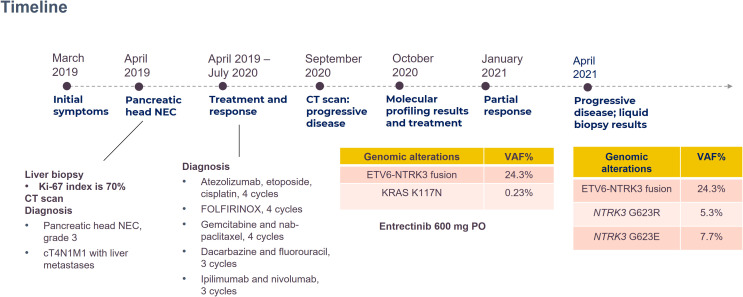
Scheme of clinical course with gene mutation tests.

The patient’s blood sample was subjected to comprehensive genomic sequencing (Foundation Medicine, Cambridge, MA, USA) to determine further treatment options. Plasma cell-free DNA (cfDNA) testing demonstrated ETV6-MTRK3 fusion, variation allele frequency (VAF) 24.3%, and Kirsten rat sarcoma vial oncogene (KRAS) K117N mutation, VAF 0.23%. The patient was prescribed entrectinib 600 mg per day. Three months later, CT revealed shrinkage of the pancreatic and liver tumors with the pancreatic tumor sized 4.2 cm and the largest hepatic lesion of 3 cm (**Figure 1B**). The patient gained 3 kg of weight per month and experienced grade 1 fatigue. Entrectinib was prescribed for another 3 months. In January 2021, a follow-up CT indicated further resolution of the pancreatic and liver lesions, sized 3.8 cm and 2.4 cm, respectively (**Figure 1C**). However, in April 2021, CT revealed a markedly increased number of liver tumors and the enlarged diameter of pancreatic tumor of 7.4 cm (**Figure 1D**). Concurrently, the patient’s total bilirubin increased from 0.87 to 8.18 mg/dL. Because of the progression of the disease, the liquid biopsy was performed again. The cfDNA analysis demonstrated the emergence of NTRK3-G623R and NTRK-G623E mutations, and the RAS mutation was undetectable (**Figure 2**). Unfortunately, the patient died during the waiting of repotrectinib, a second generation TRK inhibitor targeting acquired NTRK fusion mutations. The clinical course with genomic change identified was illustrated in **Figure 3**.

## 3 Discussion

NTRK gene rearrangements, especially fusions, result in devastating uncontrolled dimerization of the defected TRK proteins and lead to unlimited cell proliferation and growth by activating the MAPK and AKT pathways (1, 2). Among all the 295 NTRK fusions detailed in the study of Westphalen et al., ETV6-NTRK3 was the most common (26.4%) in adults, followed by TPM3-NTRK1 (21.5%) and LMNA-NTRK1 (9.5%) (1, 2). Entrectinib, a first generation TRK inhibitor, resulted in a median progression-free survival of 11 months in the integrated analysis of three phase 1–2 trials: ALKA-372-001, STARTRK-1, and STARTRK-2, which in total enrolled 54 patients with solid tumors harboring NTRK mutations (3), and further encouraging the treatment of solid tumors harboring NTRK-fusions (4, 5).

Russo et al. first reported on a patient with colon cancer who had LMNA-NTRK1 fusion genes. The patient initially exhibited a marked response to entrectinib with later development of resistance resulting from two point mutations, NTRK1 p.G595R and p.G667C, identified through liquid biopsy. Both mutations were in the coding area of ATP binding pockets (2). NTRK1 p.G595R and NTRK3 p.G523R are two of the most well-known on-target kinase domain mutations that lead to acquired resistance (6). Immunohistochemical studies of solid tumors with NTRK fusions have revealed that the fusion kinase partner might determine the subcellular localization of the fusion products and further resulted in different sensitivity to TRK inhibitors (7). Resistance following a TRK inhibitor treatment might be related to the acquisition of on-target mutations in the NTRK kinase domain of the initial oncogenic fusion and downstream bypass mechanism (8, 9). Repotrectinib (TPX-0005) is a next-generation selective anti-TRK1-3 inhibitor that is effective for treating emergent TRK mutations that failed to respond to first-generation regimens because of its smaller size and high binding ability to the ATP binding domain in preclinical studies and phase Ib/II trials (10). Repotrectinib was also granted breakthrough therapy status by the FDA in October, 2021.

Drilon et al. also described the case of a 44-year-old man with recurrent mammary analog-secretary carcinoma, whose pretreatment biopsy demonstrated ETV6-NTRK3 rearrangements, which contained an ETV6-NTRK3^G623E^ mutation. Their patient initially responded to crizotinib but the disease soon progressed despite further entrectinib and trametinib treatments. The authors further treated the patient with repotrectinib and it caused marked tumor shrinkage (10). Our patient exhibited an ETV6-NTRK3 fusion and regressive disease after entrectinib use. Nevertheless, two mutations, NTRK3-G623E and NTRK3-G623R, were identified after treatment failure. He was a potential candidate for repotrectinib treatment. We tried to apply for repotrectinib, a second generation TRK inhibitor, while the patient passed away during waiting for approval.

A KRAS mutation was also detected in our patient. Among the colorectal cancers with high microsatellite instability and NTRK-fusions reported by Westphalen et al., RAS mutation was mutually exclusive (1). Rosen et al. also demonstrated no co-occurrence of NTRK-fusions with KRAS, EGFR, BRAF, ALK, MET, or ROS-1 genes (11). On the other hand, Sigal et al. identified two patients with the co-occurrence of an NTRK1 fusion and KRAS mutation G12D and Q61R (12). One possible explanation was that the KRAS mutation in our patient may be non-functional. Another explanation is that non-fusion NTRK3 genes remarkable and resulted in normal TRK3 that eventually activated the downstream pathways.

## 4 Conclusion

To our knowledge, we have presented the first case of pancreatic NEC with a rare ETV6-NTRK3 fusion. The disease initially responded to the novel agent entrectinib, but the resistant strands of NTRK3-G623R and NTRK3-G623E later developed. K-RAS mutation co-existed but was not observed after entrectinib use. Due to the National Health Insurance Reimbursement rules, the fluorescent *in situ* hybridization (FISH) study was not done. The 2nd generation NTRK inhibitor, repotrectinib, was not on the market in Taiwan back then, so the patient passed away while waiting for the approval of compassionate use of repotrectinib. With the limitation of being an isolated case, our report still provides evidence of both the effectiveness and acquired resistance of entrectinib in treating specific NTRK rearrangement and suggests the potential role of genomic sequencing after entrectinib failure. Furthermore, liquid biopsy remains an option if acquiring tissue from the patient is challenging. The mechanisms of interaction between NTRK and RAS pathways and of the second NTRK resistance require further investigation.

## Data availability statement

The original contributions presented in the study are included in the article/Supplementary Material. Further inquiries can be directed to the corresponding author.

## Ethics statement

We explained the followings to the patient’s wife and she could fully understand that: (1) the patient’s personal information is and will be kept confidential; name and other sensitive information which might lead to the recognition of his identity will not be mentioned (2) the consent of publishing this report could be withdraw at any time without a reason before the paper is published.

## Author contributions

M-HC conceived and designed the study and contributed to the treatment planning. W-CW acquired the laboratory and clinical data and wrote the manuscript. Both authors made critical revisions and approved the final version of the manuscript.
